# Sleep state organisation of moderate to late preterm infants in the neonatal unit

**DOI:** 10.1038/s41390-022-02319-x

**Published:** 2022-12-06

**Authors:** Mary Anne J. Ryan, Sean R. Mathieson, Vicki Livingstone, Marc Paul O’Sullivan, Eugene M. Dempsey, Geraldine B. Boylan

**Affiliations:** 1 INFANT Research Centre, University College Cork, Cork, Ireland; 2 Department of Paediatrics and Child Health, University College Cork, Cork, Ireland; 3 Department of Neonatology, Cork University Maternity Hospital, Wilton, Cork, Ireland

## Abstract

**Background:**

Sleep supports neurodevelopment and sleep architecture reflects brain maturation. This prospective observational study describes the nocturnal sleep architecture of healthy moderate to late preterm (MLP) infants in the neonatal unit at 36 weeks post menstrual age (PMA).

**Methods:**

MLP infants, in the neonatal unit of a tertiary hospital in Ireland from 2017 to 2018, had overnight continuous electroencephalography (cEEG) with video for a minimum 12 h at 36 weeks PMA. The total sleep time (TST) including periods of active sleep (AS), quiet sleep (QS), indeterminate sleep (IS), wakefulness and feeding were identified, annotated and quantified.

**Results:**

A total of 98 infants had cEEG with video monitoring suitable for analysis. The median (IQR) of TST in the 12 h period was 7.09 h (IQR 6.61–7.76 h), 4.58 h (3.69–5.09 h) in AS, 2.02 h (1.76–2.36 h) in QS and 0.65 h (0.48–0.89 h) in IS. The total duration of AS was significantly lower in infants born at lower GA (*p* = 0.007) whilst the duration of individual QS periods was significantly higher (*p* = 0.001).

**Conclusion:**

Overnight cEEG with video at 36 weeks PMA showed that sleep state architecture is dependent on birth GA. Infants with a lower birth GA have less AS and more QS that may have implications for later neurodevelopment.

**Impact:**

- EEG provides objective information about the sleep organisation of the moderate to late preterm (MLP) infant.
- Quantitative changes in sleep states occur with each week of advancing gestational age (GA).
- Active sleep (AS) is the dominant sleep state that was significantly lower in infants born at lower GA.
- MLP infants who were exclusively fed orally had a shorter total sleep time and less AS compared to infants who were fed via nasogastric tube.

## Background

Of the 15 million preterm births that occur worldwide each year, >80% occur between 32 and 36 weeks GA.^1^ Current trends have shown that the rise in the rate of preterm birth is primarily attributed to the moderate to late preterm (MLP) infant group, defined as those born between 32 and 36 + 6 weeks gestation.^2^ MLP infants have a higher risk of adverse neurodevelopment in comparison to term-born infants.^3,4^

As the main behavioural state of the newborn infant, sleep is a prerequisite for normal neural network development, synaptogenesis and synaptic plasticity, particularly during the accelerated period of brain development that occurs in the last trimester.^5,6^ Sleep contributes to neural restoration, supports body repair, energy conservation and wellbeing.^7^ As sleep is an important basic need and determinant of brain development, supporting sleep and positive early life experiences for infants born during the MLP period, will support optimal neurodevelopment.^8^ Sleep in the newborn infant is comprised of active sleep (AS) (a forerunner to rapid eye movement or REM sleep), quiet sleep (QS) (a forerunner to non-rapid eye movement or NREM sleep) and indeterminate sleep (IS).

**Fig. 1:**
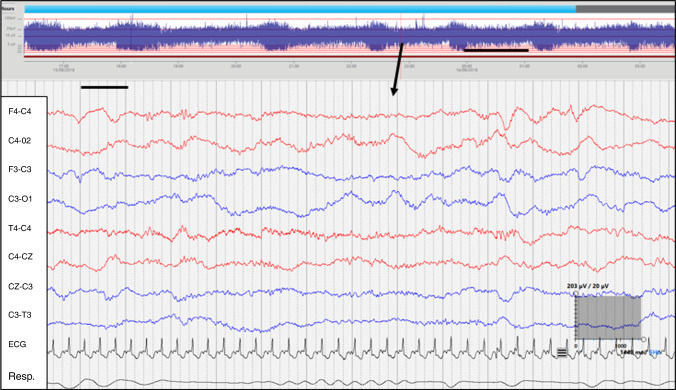
Period of continuous activity of mixed amplitude in active sleep. The black line on aEEG indicates 1 h of recording whilst the black line on cEEG indicates 1 s of recording. The arrow indicates the point on the aEEG that is displayed on cEEG. The scale of the cEEG is displayed in a grey box with amplitude measurement expressed in µV vertically and duration expressed in milliseconds horizontally.

**Fig. 2:**
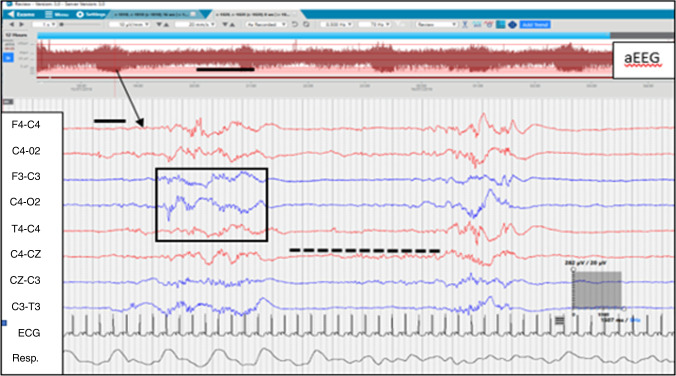
Periods of low amplitude or discontinuity (background activity <25 µV indicated by black dashed line) seen on cEEG channels in quiet sleep, with high amplitude bursts of activity, i.e., >75 µV in a black box. The black line on aEEG indicates 1 h of recording whilst the black line on cEEG indicates 1 s of recording. The arrow indicates the point on the aEEG that is displayed on cEEG. The scale of the cEEG is displayed in a grey box with amplitude measurement expressed in µV vertically and duration expressed in milliseconds horizontally.

Sleep architecture can be assessed using continuous electroencephalography (cEEG) with video.^9,10^ Each sleep state is associated with a distinct pattern of cEEG activity that alters between sleep states (Figs. 1 and 2). EEG activity describes background continuity (continuous or discontinuous), frequency of activity, and amplitude or voltage (µV) of activity, with associated alterations in behaviour and cardiorespiratory parameters (Table 1).^11–13^ Frequency of activity measured in Hertz (Hz) includes delta (0.5–4 Hz), theta (4–8 Hz), alpha (8–12 Hz) and beta (12–35 Hz) activity. Amplitude is described as either high (>50 µV), medium (between 50 and 25 µV) or low amplitude, which for the purposes of this manuscript we have identified as <25 µV.^14^

**Table 1:** Behavioural observations, cardiorespiratory parameters and EEG features identifying sleep states and wakefulness.

	Active sleep (AS)	Quiet sleep (QS)	Indeterminate sleep (IS)	Wakefulness
Behaviour	Eyes closed, may open slightly, ±REM	Eyes closed, no eye movement	Periods of opening/closing eyes	Eyes open
	Range of intermittent sporadic motor activity	Infant lying still/atonia	Slow eye movements	May be quiet, still or active
	– Low motor activity ± myoclonic twitching	May have some mouth/sucking motion	Slow startles	Fussing or crying
	– Upper and lower limb movement	May have slight startle, jerk	Intermittent sucking	High-intensity gross motor movements
	Occasional sucking movements	More difficult to rouse	Regular respirations	Irregular respirations with movement
	Quiet and low muscle tone between movements	Respirations slower, more regular than AS		Variable irregular heart rate
	Irregular shallow respiration, variable heart rate	Decreased heart rate variability than in AS		
EEG activity	Continuous activity above a threshold of 25 µV	Periods of discontinuity evident in QS (≤25 µV)		Similar to AS in quiet wakefulness
	Less than 3 s voltage attenuation <25 µV	High amplitude bursts 50–300 µV, minimum 3 s	Continuous activity high voltage (100–200 µV)	Mixed continuous activity
	Mainly high frequency >8 Hz, low/mixed amplitude (30–70 µV)	Inter-burst intervals decrease with advancing PMA		Increased artefact with movement
	Artefact with movement	Artefact with movement		Duration increasing with advancing age
aEEG activity	Lower amplitude represented by narrower portion of band	High amplitude represented by wider portion of band	Variable bandwidth	Quiet wakefulness displayed similar to AS
	Normal: a lower margin >5 µV and upper margin >10 µV	Lower margin <5 µV in QS	Not discriminated on amplitude EEG	High amplitude activity with movement

Table compiled based on findings in the literature.^11,29,32,34^

Sleep architecture undergoes quantitative and qualitative changes with maturation that is reflected in the EEG.^15,16^ Alterations in sleep architecture correlate with measurable structural changes in the brain.^17^ Cycling between sleep states requires the integration of multiple brain networks^18^. Sleep cycling is a surrogate marker of neurological wellbeing. Delayed cycling is associated with neurological dysfunction in preterm and term infants.^19,20^ cEEG provides accurate identification of changes in sleep states or cycling that is clearly evident from 30 to 32 weeks gestation. This in itself is reassuring in terms of a normal trajectory of sleep organisation and may be a useful biomarker of outcome.

There is a paucity of research on sleep architecture, particularly in the at-risk MLP infant group. We designed a prospective observational study to describe and quantify sleep of the healthy MLP infant in the neonatal unit at 36 weeks post menstrual age (PMA) (defined as birth GA plus chronological age) using cEEG with video. We hypothesised that the sleep architecture of all healthy MLP infants at 36 weeks PMA would be the same, irrespective of birth GA, sex, mode of feeding or accommodation at time of monitoring.

## Methods

This was a single-centre prospective observational study carried out in our open-plan neonatal unit in Cork University Maternity Hospital, Ireland, between July 2017 and September 2018. Ethical approval was obtained from the Clinical Research Ethics Committee of the Cork Teaching Hospitals prior to the commencement of the study.

### Participants

Clinically stable MLP infants admitted to the neonatal unit, with no chromosomal/congenital abnormalities and no neurological concerns were identified as potential participants. Whilst the development of sleep may be compromised by the morbidity of the preterm infant, all infants in this study were healthy with no physiological or neurological concerns throughout their neonatal unit stay. An invitation to participate was extended to parents whose infants satisfied the inclusion criteria. Written informed parental consent was obtained for all participating infants.

### Monitoring sleep

All infants had cEEG with video for a minimum of 12 h in the neonatal unit (pre-discharge) and as close as possible to 36 weeks but not exceeding 36 + 6 weeks PMA. We used gold standard cEEG with video to score sleep state or wakefulness based on minute-by-minute visual analysis of electrophysiological features. As heart and respiratory rate are influenced by sleep state, we also incorporated ECG (one electrode placed on each shoulder) and a respiratory sensor was secured to the abdomen with hypoallergic tape. Together these provided a visual display of alteration in heart and respiratory rate and rhythm. We did not use electrooculography (EOG) or electromyography (EMG) as we wanted to minimise the recording channels for overnight monitoring but the video was utilised to observe behaviour including ocular and facial movements. The standard of care was not altered throughout the monitoring period.

cEEG monitoring was conducted in a quiet area of the neonatal unit. To facilitate feeding schedules and parental visits, all EEG monitoring commenced between 17:00 and 20:00 h and ended the following morning between 07:30 and 10:00 h. Lifelines iEEG (Lifelines Neurodiagnostic Systems, UK) and the NicoletOne monitor (Carefusion, Wisconsin) were used for cEEG monitoring that also displayed the amplitude-integrated EEG (aEEG) as a trend of two channels above the cEEG. Whilst aEEG can be useful for the identification of sleep cycling, the information provided is a general overview of EEG activity from which it is not possible to distinguish AS from periods of quiet wakefulness and IS.^21,22^

All recordings adhered to a standard modified neonatal version of the international 10/20 system that ensured consistency in the positioning of EEG electrodes.^12^ Scalp electrode sites were prepared by cleaning with Nuprep gel and eleven sterile disposable hydrogel electrodes were applied with additional water-soluble conductive paste. Electrode placement positions were at F3, F4, C3, C4, Cz, T3, T4, O1, O2, plus a reference electrode on the frontal region of the head (R) and ground electrode (G) placed behind the left ear. An adjustable cotton hat was placed on the head over the electrodes to assist in securing electrodes overnight. cEEG monitoring commenced when all electrode impedances were <10 kOhms.

### Post-acquisition cEEG annotation

When monitoring was complete, recordings were pseudonymised and transferred to a secure server and stored for further analysis. Post-acquisition an experienced neonatal neurophysiologist (S.R.M.) reviewed EEGs for any incidental abnormal cEEG activity. Many infants exceeded 12 h of monitoring; however, only 12 h was used in the analysis (commencing with the onset of AS and as close as possible to 18:00 h). As there is a lack of consistency pertaining to the starting point of a sleep cycle in the literature,^23,24^ we defined a sleep cycle as commencing with an AS phase, with transitioning and completion of a QS phase without interruption.^24^ A complete sleep cycle may end in wakefulness or the infant may enter another phase of AS. IS occurs during the period of transition between sleep states incorporating features of AS and QS but not clearly identifiable as either.^23^ Sleep states on each cEEG recording were identified and annotated as AS (Fig. 1), QS (Fig. 2) or IS based on minute-by-minute visual analysis of cEEG activity, behavioral observation by time-locked video and cardiorespiratory parameters (see Table 1 compiled based on existing literature^9,23–25^).

Sleep interruptions that resulted in wakefulness were identified as continuous irregular or mixed EEG pattern with movement and muscle artefact, whilst cardiorespiratory observations recorded an irregular heart rate and respiration. Behavioural observations on a time-locked video provided a visual confirmation of sleep interruption and wakefulness. In quiet wakefulness, a continuous low to medium voltage mixed frequency pattern was seen with regular heart rate and respirations (Table 1).

Each 12 h cEEG with video was annotated by the same reviewer (M.A.J.R.) and a 2-h epoch of each EEG was also annotated by a second reviewer (S.R.M.). A two-way random effects intraclass correlation was calculated between the two raters on a random sample of half the EEGs. Results showed the correlation between raters for AS was 0.910 (95% CI: 0.834–0.952), QS 0.901 (95% CI: 0.817–0.947) and IS was 0.668 (95% CI: 0.448–0.812). The total duration of each sleep state was recorded, rounded to the nearest minute, and exported to SPSS.

Sleep was described as follows:

- An analysis of the duration of sleep, feeding and wakefulness over a 12-h period (sleep-wake analysis). Wakefulness incorporated periods of quiet wakefulness, time spent unsettled/fussing, ±crying.
- A total sleep time (TST) analysis, i.e., the total duration of all periods of AS, QS and IS in the 12 h recording period.
- A sleep cycle analysis described in terms of the median time in AS, QS and IS based on two overnight uninterrupted complete sleep cycles at similar time points for all infants. Cycle 1 was between 22.00hrs and 02.00hrs and Cycle 2 was between 03.00hrs and 07.00hrs. Our sleep cycle analysis was based on the median of the values obtained from the two time points.

### Statistical analysis

Statistical analysis was performed using IBM SPSS Statistics (version 25.0, IBM Corp, Armonk, NY). Continuous variables were described using the median and interquartile range (IQR) and categorical variables using frequency and percentage. The Mann–Whitney *U* test was used for comparisons of sleep parameters between two groups and the Kruskal–Wallis test was used when there were more than two groups. If statistically significant differences were found between groups for the Kruskal–Wallis test, pairwise comparisons were performed using the Mann–Whitney *U* test, with Bonferroni correction. The groups investigated were GA at birth (32–32 + 6, 33–33 + 6, 34–34 + 6, 35–35 + 6 and 36–36 + 6 weeks), sex, accommodation at the time of monitoring (cot or incubator) and mode of feeding (oral only, mixed mode). The Mann-Whitney U test was also used to investigate the relationship between GA and mode of feeding. Relationships between number of interruptions and GA and active sleep duration were investigated using Spearman’s correlation coefficient (r_s_). All tests were two-sided and a *p* value <0.05 was considered statistically significant.

## Results

Of the 117 infants recruited to this prospective observational study, 13 infants were discharged or transferred to another unit prior to cEEG monitoring, 3 infants were excluded prior to EEG recording due to suspicion of chromosomal abnormality and a further 3 excluded post EEG acquisition due to technical issues. Hence, 98 infants were included in the analysis (Table 2). Infants were from singleton (*n* = 51) and multiple pregnancies (*n* = 47) and nursed in a cot (*n* = 80) or incubator (*n* = 18) at time of monitoring. The majority of infants were exclusively fed orally (*n* = 61) whilst the remainder had either a mixed mode of feeding (*n* = 36) or were fed exclusively via nasogastric tube (*n* = 1).

**Table 2:** Demographics (*n* = 98).

	Median (IQR)
Maternal age (years)	34.5 (31.0–37.3)
Birth gestation (weeks)	34.0 (33.0–35.0)
Gestation at time of EEG (weeks)	36.1 (35.6–36.6)
Head circumference at birth (cm)	32.00 (30.75–32.90)
Apgar at 5 min	9 (9–10)
Birth weight (kg)	2.10 (1.87–2.40)
Weight at the time of cEEG (kg)	2.26 (2.02–2.47)
Postnatal age at the time of cEEG (days)	10.50 (4.75–14.00)
Sleep cycles per infant per night	6.0 (6.0–7.3)
	*n* (%)
Ethnicity—Irish	88 (89.8)
Pregnancy	
Multiple	47 (48.0)
Singleton	51 (52.0)
Sex	
Male	52 (53.1)
Female	46 (46.9)
Accommodation at time of monitoring	
Cot	80 (81.6)
Incubator	18 (18.4)
Mode of overnight feeding/infant	
All oral feeds	61 (62.2)
All nasogastric tube feeds (NGT)	1 (1.0)
Mixed feeding (oral and NGT)	36 (36.7)

*cEEG* continuous EEG.

### Sleep-wakefulness analysis

The median (IQR) of TST over the 12 h monitoring period was 7.09 h (6.61–7.76). The remaining overnight hours were spent attending to the infant’s feeding orally and periods of wakefulness. The median(IQR) duration of time spent attending to infant cares and feeding (h) was 1.58 h (1.18–2.13) and the duration of wakefulness outside this period was 3.19 h (2.38-3.85) (Table 3). As expected, infants who were feeding orally spent a significantly longer time feeding in comparison to infants who had a mixed mode of feeding (*p* = 0.004). Infants who fed exclusively orally also had a significantly longer duration of wakefulness outside of feeding (*p* = 0.033). There was no difference in feeding and wakefulness based on GA, sex or accommodation at the time of monitoring.

**Table 3:** Total sleep time, time spent feeding and awake over 12-h period (*n* = 98).

	TST(h) median(IQR)	*p* value^a^	Feeding and cares(h) median(IQR)	*p* value^a^	Wakefulness(h) median(IQR)	*p* value^a^
Overall	7.09 (6.61–7.76)		1.58 (1.18–2.13)		3.19 (2.38–3.85)	
Birth gestational age group (weeks)		0.143		0.625		0.128
32 (*n* = 19)	6.96 (6.06–7.34)		1.60 (1.34–2.06)		3.57 (2.63–4.13)	
33 (*n* = 12)	7.09 (6.84–8.01)		1.34 (1.03–1.89)		3.67 (2.45–3.96)	
34 (*n* = 36)	6.96 (6.30–7.84)		1.47 (1.12–2.36)		3.36 (2.22–3.91)	
35 (*n* = 15)	7.50 (6.91–7.82)		1.52 (1.13–2.09)		3.05 (2.07–3.29)	
36 (*n* = 16)	7.17 (6.83–8.16)		1.87 (1.13–2.20)		2.80 (2.35–3.16)	
Sex		0.606		0.177		0.586
Male (*n* = 52)	7.06 (6.59–7.73)		1.75 (1.26–2.17)		3.02 (2.29–3.90)	
Female (*n* = 46)	7.21 (6.70–7.83)		1.46 (1.10–2.13)		3.32 (2.43–3.84)	
Accommodation		0.271		0.319		0.176
Cot (*n* = 80)	7.03 (6.52–7.73)		1.53 (1.16–2.11)		3.27 (2.39–3.90)	
Incubator (*n* = 18)	7.30 (6.83–7.83)		1.87 (1.18–2.28)		2.82 (2.31–3.62)	
Mode of feeding^b^		<0.001		0.004		0.033
Oral (*n* = 61)	6.91 (6.30–7.35)		1.83 (1.32–2.26)		3.37 (2.43–3.99)	
Mixed (*n* = 36)	7.57 (6.94–8.20)		1.28 (1.09–1.71)		2.87 (2.09–3.69)	

*TST* total sleep time, *IQR* interquartile range.

^a^The Kruskal–Wallis test was used for gestational age and the Mann–Whitney *U* test was used for sex, accommodation and mode of feeding.

^b^One infant fed exclusively via NGT tube and eliminated from mode of feeding analysis (*n* = 97).

*p* < 0.05 was statistically significant.

### Total sleep time analysis

Time and percentage of time (out of 12 h) in each sleep state overall and by GA, sex, accommodation at time of monitoring and mode of feeding are presented in Table 4. AS was the dominant sleep state with a median (IQR) duration (h) of 4.58 h (3.69–5.09) of TST. The median (IQR) duration (h) of QS and IS were 2.02 h (1.76–2.36) and 0.65 h (0.48-0.89) respectively. There was a statistically significant increase in AS observed with advancing GA (*p* = 0.007). Using pairwise comparisons, we found that this difference was primarily due to the significantly lower duration of AS in the youngest birth GA group (32 weeks) compared to older GA groups of 35 weeks (adjusted *p* = 0.019) and 36 weeks (adjusted *p* value = 0.017). We investigated if the relationship between GA and AS was confounded by mode of feeding. We did not find a statistically significant relationship between mode of feeding and GA (weeks) (*p* = 0.107) and therefore, the decreased time spent in AS amongst infants born at a younger GA cannot be explained by mode of feeding. No differences were found in durations of QS (*p*=0.825) or IS (*p* = 0.544) between GA groups.

**Table 4:** Total duration of each sleep state expressed in hours and as a percentage of total sleep time (n=98).

	AS(h) median (IQR)	*p* value^a^	%AS median (IQR)	*p* value^a^	QS(h) median (IQR)	*p* value^a^	%QS median (IQR)	*p* value^a^	IS(h) median (IQR)	*p* value^a^	%IS median (IQR)	*p* value^a^
Overall	4.58 (3.69–5.09)		63.07 (56.25–67.43)		2.02 (1.76–2.36)		27.88 (24.26–34.24)		0.65 (0.48–0.89)		9.13 (6.30–12.24)	
Birth gestational age group (weeks)		0.007		0.097		0.825		0.122		0.544		0.298
32 (*n* = 19)	3.86 (3.28–4.38)		56.22 (49.43–64.79)		2.07 (1.88–2.50)		33.33 (26.63–36.00)		0.68 (0.50–0.92)		9.51 (8.36–13.59)	
33 (*n* = 12)	4.76 (4.09–5.15)		63.01 (58.49–68.29)		2.07 (1.70–2.46)		29.14 (24.68–32.77)		0.56 (0.34–0.86)		7.23 (5.31–12.92)	
34 (*n* = 36)	4.45 (3.45–5.09)		64.11 (51.20–68.33)		2.04 (1.78–2.23)		27.65 (23.39–34.84)		0.68 (0.48–0.94)		9.86 (6.96–12.61)	
35 (*n* = 15)	4.87 (4.60–5.08)		64.94 (60.49–66.86)		1.98 (1.70–2.35)		26.19 (22.43–30.65)		0.65 (0.57–0.87)		8.87 (6.51–11.73)	
36 (*n* = 16)	4.95 (4.14–5.48)		65.91 (57.62–69.35)		1.92 (1.43–2.55)		26.15 (21.53–34.21)		0.56 (0.37–0.87)		7.16 (5.44–12.26)	
Sex		0.457		0.200		0.089		0.129		0.845		0.825
Male (*n* = 52)	4.59 (3.70–5.24)		64.55 (55.40–69.52)		1.95 (1.71–2.28)		26.55 (23.08–33.65)		0.64 (0.48–0.88)		8.43 (6.06–13.38)	
Female (*n* = 46)	4.40 (3.68–5.06)		61.76 (56.25–65.73)		2.14 (1.88–2.49)		29.59 (25.84–35.09)		0.65 (0.50–0.91)		9.23 (6.78–11.69)	
Accommodation		0.790		0.335		0.073		0.263		0.989		0.869
Cot (*n* = 80)	4.49 (3.70–5.15)		63.78 (56.23–68.22)		1.99 (1.71–2.31)		27.88 (23.62–34.09)		0.65 (0.48–0.88)		9.23 (6.38–12.26)	
Incubator (*n* = 18)	4.75 (3.60–4.98)		60.98 (55.44–65.10)		2.24 (1.93–2.51)		27.88 (26.18–36.19)		0.60 (0.49–0.93)		8.43 (6.03–12.35)	
Mode of feeding^b^		<0.001		0.433		0.291		0.473		0.852		0.229
Oral (*n* = 61)	4.29 (3.45–4.86)		63.25 (52.19–68.05)		2.01 (1.75–2.33)		27.90 (24.62–34.66)		0.473 (0.47–0.88)		9.68 (6.42–12.81)	
Mixed (*n* = 36)	4.94 (4.47–5.32)		63.09 (59.31–67.01)		2.04 (1.73–2.52)		27.65 (22.76–30.92)		0.59 (0.51–0.93)		8.01 (6.04–11.49)	

The percentage (%) for each infant is the percentage of time spent in each sleep state.

*AS* active sleep, *QS* quiet sleep, *IS* indeterminate sleep, *IQR* interquartile range.

^a^The Kruskal–Wallis test was used for gestational age and the Mann–Whitney *U* test was used for sex, accommodation and mode of feeding.

^b^One infant fed exclusively via NGT tube and eliminated from mode of feeding analysis (*n* = 97).

*p* < 0.05 was statistically significant.

In a previous study of this MLP group (*n* = 98), we found that 23.3% of overnight sleep cycles in an MLP infant group were interrupted, almost exclusively for the purposes of feeding.^26^ Therefore, we investigated if this increase in AS with increasing GA could have been due to a difference in the number of sleep interruptions that are followed by a period of wakefulness and then a recommencement of AS. The number of interruptions increased with increasing GA (r_s_ = 0.20, *p* = 0.048) but no association was found between number of interruptions and duration of AS (r_s_ = 0.05, *p* = 0.593). Thus, number of interruptions was not a confounder of the association between GA and AS. The total duration of AS did not significantly differ based on sex or accommodation (*p* = 0.457 and 0.790 respectively).

There were no differences in duration of QS based on sex, accommodation and mode of feeding *p* = 0.089, 0.073 and 0.291, respectively) or IS based on the same groupings *p* = 0.845, 0.989 or 0.852, respectively.

### Sleep cycle analysis

All infants displayed sleep cycling on cEEG with a median (IQR) of 6.0 (6.0–7.3) uninterrupted sleep cycles throughout the night. Sleep cycle duration and the duration of AS, QS and IS within, are described by GA, sex, accommodation at time of monitoring and mode of feeding are presented in Table 5. The median (IQR) duration (min) of an overnight uninterrupted sleep cycle was 37.00 min (32.00-45.13) with a median(IQR) of 16.50 min (12.50-22.50) for AS, 16.75 min (14.00-19.50) for QS and 2.75 min (2.00-3.50) for IS.

**Table 5:** Sleep cycle duration overall by sleep state/groups including feeding and wakefulness (*n* = 98).

	Sleep cycle duration(min) median(IQR)	*p* value^a^	AS duration median(IQR)	*p* value^a^	QS duration(min) median(IQR)	*p* value^a^	IS duration(min) median(IQR)	*p* value^a^
Overall	37.00 (32.00–45.13)		16.50 (12.50–22.50)		16.75 (14.00–19.50)		2.75 (2.00–3.50)	
Gestational age per group (weeks)		0.105		0.247		0.001		0.210
32 (*n* = 19)	40.50 (31.00–50.00)		15.00 (9.00–23.00)		19.50 (17.00–22.50)		3.00 (2.00–4.00)	
33 (*n* = 12)	39.00 (31.13–43.75)		16.50 (13.00–22.00)		17.75 (16.00–19.00)		2.00 (1.63–3.00)	
34 (*n* = 36)	34.50 (30.00–39.00)		14.25 (12.00–21.38)		15.00 (11.63–18.38)		2.75 (2.00–3.38)	
35 (*n* = 15)	39.00 (35.00–45.00)		19.00 (15.00–23.00)		16.00 (15.00–20.00)		2.00 (1.50–3.00)	
36 (*n* = 16)	41.50 (35.13–48.75)		20.00 (15.00–25.88)		15.00 (13.00–18.00)		2.75 (1.63–3.38)	
Sex		0.706		0.591		0.151		0.294
Male (*n* = 52)	37.00 (32.00–44.50)		16.75 (13.00–23.00)		16.00 (13.00–19.38)		3.00 (2.00–3.50)	
Female (*n* = 46)	38.00 (32.00–46.50)		16.25 (9.88–22.13)		17.25 (15.00–19.63)		2.50 (2.00–3.13)	
Accommodation		0.151		0.451		0.666		0.386
Cot (*n* = 80)	36.75 (32.00–44.25)		15.50 (12.63–22.00)		16.50 (14.00–19.50)		2.50 (2.00–3.00)	
Incubator (*n* = 18)	43.00 (33.50–48.25)		21.25 (11.25–24.63)		18.00 (14.63–19.63)		3.00 (1.88–4.00)	
Mode of feeding^b^		0.098		0.143		0.363		0.820
Oral (*n* = 61)	37.00 (31.50–44.00)		15.00 (11.50–22.25)		17.00 (13.25–20.00)		2.50 (2.00–3.50)	
Mixed (*n* = 36)	39.50 (34.63–46.50)		19.50 (13.25–23.75)		16.25 (14.25–18.00)		2.75 (2.00–3.00)	

*AS* active sleep, *QS* quiet sleep, *IS* indeterminate sleep, *IQR* interquartile range.

^a^The Kruskal–Wallis test was used for gestational age and the Mann–Whitney *U* test was used for sex, accommodation and mode of feeding.

^b^One infant fed exclusively via NGT tube and eliminated from mode of feeding analysis (*n* = 97).

*p* < 0.05 was statistically significant.

Within sleep cycles, QS duration differed by GA group (*p* =0.001). Pairwise comparisons revealed significant differences between the youngest GA group (32 weeks) and the older GA groups of 34 weeks (adjusted *p* = 0.001) and 36 weeks (adjusted *p* = 0.010). The duration of AS, IS or sleep cycle duration did not differ between GA groups (*p* = 0.247, 0.210 and 0.105 respectively). There was no difference in the sleep cycle duration or the duration of AS, QS, IS within uninterrupted sleep cycle based on sex, accommodation or mode of feeding.

## Discussion

In this study, we used cEEG with video to quantify the overnight sleep of a large cohort of healthy MLP infants in the neonatal unit. At the outset, it must be considered that the genetically defined pre-programmed pathway of normal sleep ontogenesis may be altered or supported through physiological and sensory environmental factors.^27,28^ Stressors within the neonatal unit may compromise the quality and quantity of sleep.^11,29,30^

As the primary activity of the developing brain, sleep plays a crucial role in brain development. As the dominant sleep state of the MLP group, AS provides endogenous stimulation facilitating neural network maturation, synapse formation and synaptic plasticity. AS supports memory, learning, emotional and motor development and has a strong correlation with brain growth.^6^ It is also a prerequisite state for psychosocial and normal sensory system development.^31^ A reduction in AS in the preterm infant may reduce the efficiency of information transmission and decrease overall brain volume.^32,33^ QS is a prerequisite for maturation, supporting connectivity between networks and the preservation of brain plasticity.^8^ QS is also associated with cognitive function and the pre-consolidation phase of learning, memory and the maintenance of physiological homoeostasis.^34,35^

The duration of overnight sleep was shorter than expected whilst the duration of wakefulness was longer in this MLP group. Interestingly, infants born in the lower GA groups had the longest duration of wakefulness which may suggest an increased level of maturation. However, these differences did not reach a level of significance (*p* = 0.128). We have shown that in the MLP infant group the median (IQR) duration of time spent sleeping was 7.09 h (6.61–7.76 h) or 59.1% (55.1–64.7%) of 12 hr monitoring period. It could be considered that 14.18 h extrapolated over a 24-h period falls short of the expected average of 16–18 h of sleep for newborn infants.^35,36^ Intuitively this might be expected to be an overestimate of TST as an increase in clinical activity and sensory exposure by day may lead to infants sleeping less during daytime hours. However, Ardura et al. suggested the opposite to be true, finding that preterm infants at 35 weeks GA (*n* = 11) slept for 8.88 h during day time hours (08.00–20.00 h) and 8.22 h overnight (20.00–08.00 h). At 36 weeks GA (*n* = 14) infants slept for 8.96 h by day and 7.51 h overnight.^37^

When investigating the effects of handling on sleep in late preterm low birth weight infants nursed in an incubator (*n* = 12), Maki et al. found no significant difference in duration of AS, QS, IS or wakefulness between day and night time sleep (*p* = 0.83, 0.84, 0.73 and 0.99, respectively).^38^ These authors also observed that the infant group under study slept for approximately 13.7 h of the 24-h period, i.e., 57.2% of 24 h. As we found no significant difference in TST between infants nursed in a cot or an incubator (*p* = 0.271), these findings are comparable to ours. Sleep duration recommendations have been considered to be based on weak scientific evidence.^39^ The preterm infant may spend up to 90% of the day sleeping which decreases with advancing GA.^40^ Whilst 14–17 h are identified as appropriate for term newborn any duration between 11 and 19 h has been considered acceptable.^39^

In our TST analysis, we found that the majority of overnight sleep was AS, representing 63.07% (56.25–67.43%) of the TST. As sleep in the newborn begins with AS it is not surprising that overall, TST was dominated by AS, as following each sleep cycle interruption (or spontaneous awakening) a new phase of AS sleep must begin. It is interesting that at 36 weeks, infants born in the lower GA groups had the longest duration of wakefulness in comparison to those born at a higher GA. Perhaps this suggests an increased maturity amongst infants born at a lower GA. However, these differences did not reach a level of significance (*p* = 0.128).

The overall duration of AS was significantly lower amongst infants born in a lower GA group (*p* = 0.007), whilst the duration of QS was longer differences were not statistically significant (0.825). Consistent with other studies, our results support a trend towards changes in sleep organisation with advancing GA. EEG maturation occurs with increased chronological age, i.e., a decrease in the overall time spent in AS and an increase in QS (Table 4).^19,41,42^ However, there is some conflicting evidence suggesting the opposite trajectory may be true. Han et al. utilised cEEG, aEEG, EOG, EMG over a 4-h period and described the changes that occurred with advancing GA. In comparing the percentage of each sleep state between a 34–35 GA group (*n* = 20) and a 36–37 GA group (*n* = 23), results showed an increase in the percentage of AS sleep duration from 39.8 to 46.6%, a decrease in the percentage of QS duration from 36.8 at to 32.3%, a decrease in IS from 7.9 to 5.3%, whilst duration of wakefulness differed minimally 15.5–15.8% between groups.^9^ In a review of sleep state measurement, Werth et al. described a plateau in AS duration between 30 and 36 weeks GA at 60–65% of sleep time,^43^ which is similar to our results of a median of 63.07%. QS was described as increasing until 36 weeks to approximately 38% that is higher than our findings of a median of 27.88%. Thereafter, Werth et al. described both sleep states decrease as wakefulness increases. Similar to Foreman et al. we found no disparities based on sex in our analysis of sleep state duration.^44^

In our analysis of uninterrupted sleep cycles, the median (IQR) duration was 37.00 min (32.00–45.13) that is shorter than previously reported for MLP infants at 36 weeks PMA.^23,24,45^ Learning and memory are dependent on normal cycling.^46^ Our results showed that the duration of QS within an uninterrupted sleep cycle differed by GA age group only (*p* = 0.001) with the younger birth GA group having a significantly longer QS period. Our sleep cycle analysis showed differences in the duration of AS by GA group within an uninterrupted sleep cycle but these differences did not reach statistical significance.

There is extensive variability in the literature pertaining to the duration of a sleep cycle in preterm infants at 35–36 weeks PMA. Tsuchida et al. reported a sleep cycle duration of 50–65 min for infants >35 weeks PMA.^24^ Curzi-Dascalova et al. described the sleep cycle duration of a small group of participants (*n* = 11 between 35 and 36 weeks GA) by the mean +/− standard error of the mean as 69.4 +/− 6.1 min.^45^ Even for healthy term-born infants, sleep cycles typically last a mean of 50–60 min with a range of 30–70 min described.^47^ Dereymaeker et al. also report extensive variability in cycle duration amongst infants of 35–36 weeks GA that ranged from 6.3 to 109 min with a mean (SD) of 50.9 min (19.2 min).^48^ In our methodology we defined a sleep cycle and its starting point.^24^ However, inconsistencies in sleep cycle definitions were noted.^23,24^ Some studies did not define it but described sleep cycling in broad terms such as fluctuations between sleep states.^49^

Heterogeneity in methodology is evident in the literature making it difficult to compare like with like. Many sleep studies carried out have been based on behaviour observation, with or without the inclusion of ECG, EMG and EOG,^35,37,44,50^ or sleep state determined based on activity or actigraphy.^18^ Whilst some studies did utilise cEEG with video, differences in inclusion/exclusion criteria, duration and timing of monitoring and environmental conditions were evident.^9,36,45,51^ Whilst environmental factors may impact the duration and intensity of sleep, intra-individual variation in sleep organisation may also be attributed to endogenous factors with genetic links.^44,52^ To date, the majority of studies that used cEEG to monitor preterm sleep have been during daytime hours and recording duration has rarely been longer than 4 h. Shorter duration of monitoring may not be truly representative of sleep cycle duration for an infant group. Inclusion/exclusion criteria also varied with little information provided in relation to mode of feeding and the sleeping environment.^9,50,51,53^ To the best of our knowledge this is the first study to date to utilise cEEG with video over 12 h to determine sleep state organisation of MLP infants at 36 weeks PMA. All infants in this study were cared for under similar environmental conditions and without disturbances for clinical purposes.

Infant feeding in our unit is time-based, generally 3 hourly for infants learning to feed or 4 hourly when larger volumes are tolerated orally. Infants that had a mixed mode of feeding overnight had a longer TST (*p* < 0.001). This is the first study to report these findings. With respect to guidelines for establishing feeding in preterm infants we use passive tube feeds combined with oral feeds offered on an individualised basis. Allowing the infant to sleep and grow through passive feeding supports neurodevelopment and consequently the development of the infants suck swallow coordination. When TST in both mode of feeding groups was broken down by sleep state, the more immature infants who were partially tube fed had a longer AS duration in comparison to infants who fed exclusively orally (*p* < 0.001). Achieving a balance between supporting the infant in establishing feeding and protecting sleep is key.

There is much we do not understand about sleep. Theunissen et al. proposed that the exact role of sleep in brain development is unknown suggesting that perhaps they may be only temporally related, or they are independent, or one may drive the other.^54^ We do not know what factors are particularly destructive to infant sleep architecture and brain development. However, there is a general consensus that an environment supporting the preservation of good quality sleep without disturbance will strengthen sleep architecture and support optimal brain development.^10,55^

This is the largest study to date focusing on the sleep of the healthy uncomplicated MLP infant through overnight cEEG monitoring with video carried out at a similar PMA under similar environmental conditions. These factors reduced the risk of sleep being compromised by morbidity or environmental exposure during EEG monitoring. The number of male and female participants was reasonably balanced as was the number of multiple and singleton pregnancy’s, removing bias related to these factors in the study. We also identified mode of feeding for each infant, a factor that has not been widely reported in previous sleep studies. Previous EEG sleep studies have based percentages of each sleep state on the interpretation of a single sleep cycle or short periods of cEEG.^23,24,43,56,57^

This study also had some limitations. Our study was conducted in an open-plan neonatal unit where we aimed to minimise environmental stimuli by caring for infants in a quiet low-lit area of the unit during monitoring. However, we did not measure ambient sound or control for potential confounding factors such as environmental temperature, sleeping position or clinical workload. We did not use EOG like many studies but we did use video to assess overall behavioural observations including ocular and facial movements.

As variations in infant sleep play an important role in cognitive development and physical growth,^58^ future sleep research incorporating sequential and prolonged cEEG with video should be considered the gold standard. This will provide an objective robust estimate of TST and a greater insight into sleep architecture unique to GA. It will also provide confirmation of achievement of sleep state organisation appropriate for GA which may be a potential biomarker of future outcome.^57,59^ Alteration in EEG sleep organisation or the maturation trajectory may be an expression of brain dysfunction.^60^ Further research is also required to assess any associations between sleep variables and long-term developmental outcomes.^61^

Sleep staging in preterm infants is complex, labour intensive and requires expertise. A growing body of researchers are using machine learning to design algorithms such as seizure detection algorithms.^62^ Sleep detection algorithms, detect specific features of sleep,^63^ automate sleep staging,^64^ and estimate brain maturity.^65^ Automated approaches have shown great promise in providing robust quantitative data pertaining to sleep organisation and perhaps identifying future biomarkers of developmental outcome. Identifying infants who warrant ongoing surveillance and early intervention will potentially improve outcome, which is the ultimate aim of neonatal care.

## Conclusion

This is the first study to describe overnight 12-h sleep architecture in a large homogenous group of MLP infants using cEEG with video. Our findings provide robust normative data for each week of GA unique to the healthy MLP infant group. Quantitative changes in sleep state may be used as a reference point in future studies and a potential biomarker of developmental outcome.

## Data Availability

For this current study, it is not possible to share data sets. The clinical data are collected under written proxy consent from the participants' guardians/parents that did not include permission for sharing or open data. To be allowed to share these data under Irish Health Research Regulations we are required to re-consent families or obtain approval from the Health Regulation Consent Declaration Committee.
